# The Effect of Maternal Exposure to Air Pollutants and Heavy Metals during Pregnancy on the Risk of Neurological Disorders Using the National Health Insurance Claims Data of South Korea

**DOI:** 10.3390/medicina59050951

**Published:** 2023-05-15

**Authors:** Kuen Su Lee, Won Kee Min, Yoon Ji Choi, Sejong Jin, Kyu Hee Park, Suhyun Kim

**Affiliations:** 1 Department of Anesthesiology and Pain Medicine, Eulji University Uijeongbu Eulji Medical Center, Eulji University School of Medicine, Uijeongbu 11759, Republic of Korea; dlrmstnx@eulji.ac.kr; 2 Department of Anesthesiology and Pain Medicine, Korea University Ansan Hospital, Korea University College of Medicine, Ansan 15355, Republic of Korea; 3 Department of Neuroscience, Korea University College of Medicine, Seoul 02841, Republic of Korea; holicer@korea.ac.kr; 4 Department of Pediatrics, Korea University Ansan Hospital, Ansan 15355, Republic of Korea; czrabbit@korea.ac.kr; 5 Department of Biomedical Sciences, College of Medicine, Korea University, Seoul 02841, Republic of Korea; dieslunae@korea.ac.kr

**Keywords:** air pollution, autism spectrum disorder, epilepsy, heavy metals

## Abstract

The objective of this study was to evaluate the effects of high levels of maternal exposure to ambient air pollution and heavy metals on risks of autism spectrum disorder (ASD) and epilepsy using the National Health Insurance claims data of South Korea. The data of mothers and their newborns from 2016 to 2018 provided by the National Health Insurance Service were used (*n* = 843,134). Data on exposure to ambient air pollutants (PM2.5, CO, SO_2_, NO_2_, and O_3_) and heavy metals (Pb, Cd, Cr, Cu, Mn, Fe, Ni, and As) during pregnancy were matched based on the mother’s National Health Insurance registration area. SO_2_ (OR: 2.723, 95% CI: 1.971–3.761) and Pb (OR: 1.063, 95% CI: 1.019–1.11) were more closely associated with the incidence of ASD when infants were exposed to them in the third trimester of pregnancy. Pb (OR: 1.109, 95% CI: 1.043–1.179) in the first trimester of pregnancy and Cd (OR: 2.193, 95% CI: 1.074–4.477) in the third trimester of pregnancy were associated with the incidence of epilepsy. Thus, exposure to SO_2_, NO_2_, and Pb during pregnancy could affect the development of a neurologic disorder based on the timing of exposure, suggesting a relationship with fetal development. However, further research is needed.

## 1. Introduction

Air pollution is a major risk factor for global health. Exposure to air pollution has been linked to increased mortality and morbidity, contributing significantly to the overall global disease burden [1]. Globally, the number of all-cause deaths from overall air pollution increased by 2.62% from 1990 to 2019 [2]. The Health Effects Institute’s State of Global Air reported that PM2.5, a type of fine-particulate air pollution, is the sixth-highest risk factor for death globally, accounting for approximately four million deaths in 2019 alone [3]. Over 90% of the world’s population live in areas where the air quality standards set by the WHO are not met. In Asian megacities, air pollution concentrations have been observed to be the highest worldwide. Recently, Korea has undergone rapid economic growth, and the air quality has worsened.

Air pollution is caused by complex components including nitrogen dioxide (NO_2_), sulfur dioxide (SO_2_), and particulate matter (PM). NO_2_, SO_2_, PM, and indirectly generated ozone (O_3_) are major air pollutants that are related to exhausts from vehicles and industrial energy consumption, which are caused by urbanization and industrialization [4,5,6,7].

Generally, heavy metals are amalgamated with PM [8] and mainly originate from diesel and gasoline exhaust fumes from local traffic and industrial areas [9,10].

Air pollution is known to affect mortality and the prognosis of cardiovascular disease, respiratory disease, and stroke [11,12,13]. Recent studies have focused on the potential effects of exposure to NO_2_, SO_2_, and PM2.5, which showed that a maternal exposure factor that can adversely affect even the prenatal period [14,15].

PM2.5, with various deleterious components, can enter the blood circulation through the lungs [16,17]. In addition, PM2.5 exposure during pregnancy can induce oxidative stress and an inflammatory response. It can affect the fetus through changes in the uterine environment and placental function [18,19]. The prenatal period is critical for brain development. It is a complicated process determined by both genetic and external factors. Deleterious factors during the prenatal period might have severe and long-term adverse effects on brain structure and function, resulting in neurodevelopmental disorders.

Some studies have shown that exposure to NO_2_, SO_2_, and PM2.5 is a risk factor for stillbirth and spontaneous abortion, supporting the notion that exposure to NO_2_, SO_2_, and PM2.5 can affect the fetus [20,21]. Regarding neurodevelopment, it is known that PM2.5 can induce oxidative stress and an inflammatory response [8] and that both oxidative stress and an inflammatory response can affect the expression of brain-derived neurotrophic factor (BDNF) and cyclic AMP response element-binding protein (CREB), which are well-known neurodevelopment factors [22,23,24]. Some studies have demonstrated that exposure to PM2.5 is related to changes in the expression of BDNF and CREB [25,26,27].

The etiology of autism spectrum disorder (ASD) and epilepsy is still not fully known so far. They were once regarded as genetic diseases [28,29,30]. However, some studies have reported that both genetic and environmental factors can contribute to these diseases [31,32,33]. Therefore, the objective of this study was to evaluate whether high levels of maternal exposure to NO_2_, SO_2_, and PM2.5 could increase the risk of neurological disorders such as autism spectrum disorder and epilepsy using the National Health Insurance claims data of South Korea.

## 2. Materials and Methods

### 2.1. Study Population

This study was approved by the Institutional Review Board of the Korea University Ansan Hospital (2021AS0317). Information obtained from the Korean National Health Insurance (NHI) claims database from January 2016 to December 2020 was used in this study. The NHI claims database provides information on all the insurance claims of the Korean population.

This study cohort included NHI claims data for babies with short gestation periods and low birth weights (“P07”), comprising singletons and twins (“Z38.0–Z38.5”) born in hospitals, and mothers who gave birth from January 2016 to December 2018. Multiple births and missing data were excluded, as they could have affected the results of this study.

The baseline characteristics, underlying diseases, and follow-up data of study subjects were extracted from the NHI claims database. The measured data on ambient air pollutants (PM2.5, CO, SO_2_, NO_2_, and O_3_) and heavy metals (Pb, Cd, Cr, Cu, Mn, Fe, Ni, and As) in South Korea from January 2015 to December 2018 were extracted from the Korea Environment Corporation (https://www.airkorea.or.kr/eng/ accessed on 18 April 2022.). The atmospheric conditions data were matched to mothers and their newborns based on the mother’s NHI registration area. Air pollutant measurement data measured during pregnancy were matched based on the mother’s health insurance claim registration area. The gestation period was divided into three stages. The first 1–3 months were defined as stage 1, 4–7 months as stage 2, and 8–10 months as stage 3. In the case of household income, the bottom 40% was defined as low and the top 5% was defined as high. Premature (“P07.2–F07.3”) and twin (Z38.3–Z38.5) codes were used.

Autism spectrum disorder (“F84.0–F84.9”), excepting Rett’s syndrome (F84.2), and epilepsy (“G40.0–G40.9”) were the disease codes used in this study. The minimum observation period for infants up to disease onset was maintained as two years or more.

### 2.2. Statistical Analysis

Data are presented as the mean ± standard deviation and number (%) of patients. The confounding variables and essential characteristics of the groups with and without autism spectrum disorder and groups with and without epilepsy were analyzed using an independent t-test for continuous variables and Fisher’s exact test or the chi-square test for categorical variables.

Logistic regression was performed, and odds ratios and 95% CIs for autism spectrum disorder or epilepsy adjusted for maternal age, education, infant sex, gestational season, and household income were analyzed for air pollutant and heavy metal exposure according to pregnancy stage in a single-pollutant model.

For autism spectrum disorder, two air pollutants and two heavy metals were selected according to forward selection with a four-pollutant model. After adjusting for maternal age, education level, infant sex, pregnancy period, and household income, the odds ratios and 95% CIs for autism spectrum disorder were analyzed based on exposure to air pollutants and heavy metals according to pregnancy stage.

For epilepsy, two air pollutants and three heavy metals were selected according to forward selection with a five-pollutant model. After adjusting for maternal age, education level, infant sex, pregnancy period, and household income, odds ratios and 95% CIs for epilepsy were analyzed based on exposure to air pollutants and heavy metals according to pregnancy stage. Additionally, adjusted odds ratios and 95% CIs for the neurologic disorders and exposure to air pollutants and heavy metals according to months of pregnancy in a one-pollutant model were obtained.

All statistical analyses were performed using SAS^®^ ver. 9.4 (Statistical Analysis Software 9.4, SAS Institute Inc., Cary, NC, USA). Differences were considered statistically significant if the *p*-value was less than 0.05.

## 3. Results

**Figure 1:**
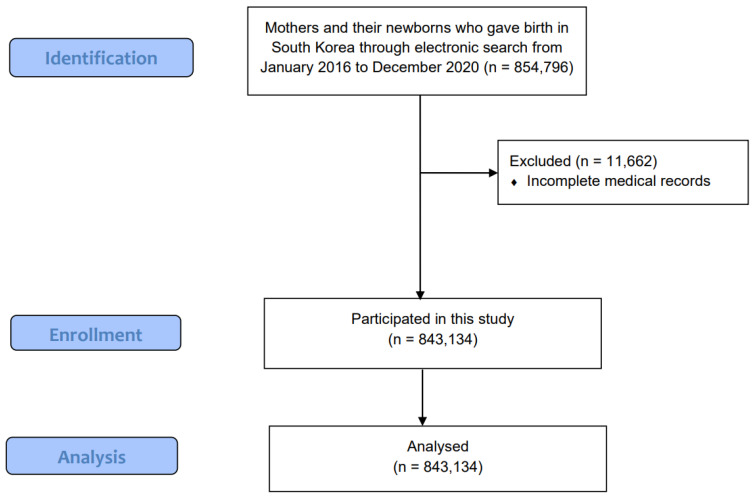
Consort flow diagram of this study.

The medical records of 854,796 mothers from January 2016 to December 2020 and their newborns from January 2016 to December 2018 were reviewed (Figure 1). There were 5493 infants with autism spectrum disorder and 3190 infants with epilepsy. A total of 11,662 patients were excluded due to incomplete medical records. Finally, 843,134 patients were included in this study.

Table 1 shows the demographic and prenatal characteristics of the study subjects. For the infants with ASD, the proportion of maternal ages of 40 years or older was higher (23.5%) compared to that for infants without ASD (18.5%, *p* < 0.001). There were more males (71.6%) among the infants with ASD than the infants without ASD (51.25%, *p* < 0.001). Furthermore, more infants with ASD were born in winter (33.7%, *p* < 0.001), and these infants included more preterm babies (15.1%) and twins (3.5%, *p* < 0.001). For infants with epilepsy, the proportion of maternal ages over 40 was higher (23.9%) than that for infants without ASD (18.5%, *p* < 0.001) There were more males (55.3%) among the infants with epilepsy than the infants without epilepsy (51.3%, *p* < 0.001). Infants who were born in winter (36.7%) (*p* < 0.001) and those who were preterm (13.5%) were more prevalent among the infants with epilepsy than the infants without epilepsy (*p* < 0.001).

**Figure 2:**
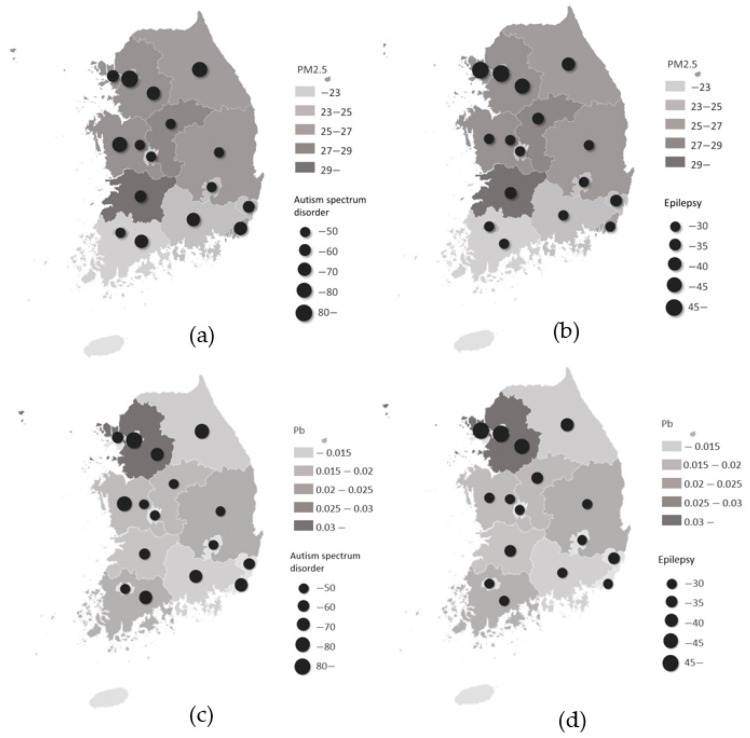
Spatial distribution of the mean PM2.5 and NO_2_ concentrations and the number of neurologic disorders in South Korea. (**a**) PM2.5 and autism spectrum disorder, (**b**) PM2.5 and epilepsy, (**c**) Pb and autism spectrum disorder, (**d**) Pb and epilepsy. Data are presented as the mean and number of patients.

Table 2 shows the summary statistics of air pollutants and heavy metals by case. Figure 2 shows the spatial distribution of the mean PM2.5 and NO_2_ concentrations and the number of neurologic disorders in South Korea. An increase in the mean PM2.5 or Pb was associated with an increase in the incidence of ASD and epilepsy in newborns.

The adjusted odds ratios and 95% CIs for autism spectrum disorder and epilepsy and exposure to air pollutants and heavy metals according to the stage of pregnancy in the single-pollutant model are shown in Table 3.

The adjusted odds ratios and 95% CIs for autism spectrum disorder and exposure to air pollutants and heavy metals according to the stage of pregnancy in a four-pollutant model are shown in Table 4. The adjusted odds ratio and 95% CIs for epilepsy and exposure to air pollutants and heavy metals according to the stage of pregnancy in a five-pollutant model are shown in Table 5.

ASD was associated with increased total mean concentrations of SO_2_, NO_2_, and Pb during the entire pregnancy. SO_2_ (OR: 2.723, 95% CI: 1.971–3.761) and Pb (OR: 1.063, 95% CI: 1.019–1.11) were strongly associated with the incidence of ASD when infants were exposed to them in the third trimester of pregnancy. Epilepsy was associated with increased total mean concentrations of SO_2_ and NO_2_ during the entire pregnancy. Pb (OR: 1.109, 95% CI: 1.043–1.179) in the first trimester of pregnancy and Cd (OR: 2.193, 95% CI: 1.074–4.477) in the third trimester of pregnancy were also associated with the incidence of epilepsy.

**Figure 3:**
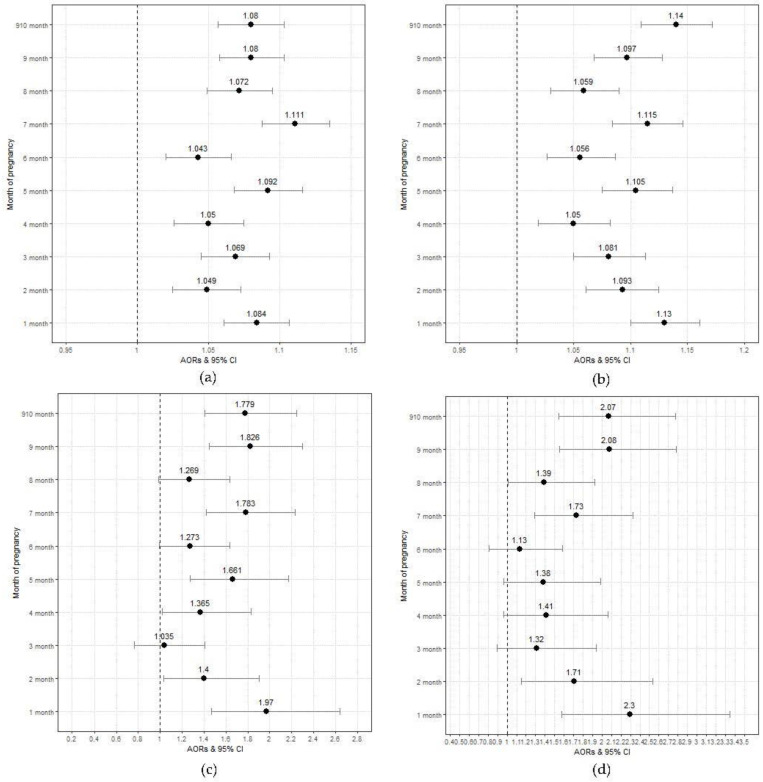
Adjusted odds ratios and 95% Cis for neurologic disorders and exposure to air pollutants and heavy metals by months of pregnancy in a one-pollutant model. (**a**) Pb and autism spectrum disorder by months of pregnancy, (**b**) Pb and epilepsy by months of pregnancy, (**c**) Cd and autism spectrum disorder by months of pregnancy, and (**d**) Cd and epilepsy by months of pregnancy. Logistic regression model adjusted for maternal age, education, infant sex, season of conception, and household income.

Figure 3 shows the adjusted odds ratios and 95% CIs for the neurologic disorders and exposure to air pollutants and heavy metals according to months of pregnancy in a one-pollutant model. The association between the concentration of Pb by pregnancy period and the occurrence of ASD and epilepsy increased over the entire pregnancy period. On the other hand, Cd had a closer association with the occurrence of ASD and epilepsy in the early and late pregnancy periods.

## 4. Discussion

This study demonstrated that exposure to high concentrations of SO_2_, NO_2_, and Pb during the entire pregnancy period was associated with the development of ASD or epilepsy. The biological mechanism linking in utero exposure to air pollution and neural development in children is not yet fully understood. Maternal exposure to air pollution, including NO_2_/SO_2_ or PM2.5 and heavy metals, can lead to inflammation, oxidative stress, and DNA methylation placenta via lung tissues [34,35,36,37,38]. Oxygen and nutrient transport to the fetus are then disturbed, and inflammatory cytokines from the maternal circulation might be transported to the fetus, causing a fetal systemic inflammatory response which can adversely affect fetal development [34,35,36,39,40,41]. The fetal period is a critical window for brain development. Air pollutants in utero can significantly increase the susceptibility of infants to neurological diseases after birth [42].

This study demonstrated that exposure to high concentrations of SO_2_ during the third trimester was associated with the birth of children with ASD. This study also revealed that exposure to SO_2_ during the early and late pregnancy periods was associated with the birth of a child with epilepsy. Regarding ASD, a previous study showed no association between the level of prenatal SO_2_ exposure and the risk of ASD [43]. In that study, the average exposure level of SO_2_ during pregnancy was approximately 5.8 ppb during the observation period, which is similar to the result of our study (an average of 5 ppb), but the relationship between prenatal SO_2_ exposure and the risk of ASD showed a different result. Other studies have reported that prenatal SO_2_ exposure is associated with poor or impaired neurodevelopment in early childhood [44,45]. It is known that SO_2_ may lead to neurotoxicity by inducing oxidative stress, DNA damage, and apoptosis, as well as DNA methylation [46,47,48,49,50]. Previous studies on the absorption, distribution, and retention of SO_2_ in mammalian subjects have indicated that sulfur can be absorbed into the blood circulation and transported to the central nervous system [51,52]. When SO_2_ reaches the CNS, its biochemical effects can change the enzymatic activities of the CNS [53]. SO_2_ can depress some enzymatic activities of glucose metabolism [54]. With respect to prenatal exposure, Choi et al. [50] reported that prenatal DNA-methylation-associated SO_2_ exposure is associated with an increased ADHD rating scale in later childhood. Moreover, Liu et al. reported that prenatal SO_2_ exposure is positively related to the fetal hs-CRP level, a biomarker of systemic inflammation, and Liu et al. also suggested that prenatal SO_2_ exposure might interfere with fetal glucolipid metabolism by inducing fetal systemic inflammation [55]. However, the study reported no significant association between NO_2_ exposure and increased hs-CRP levels, indicating that the mechanisms through which SO_2_ and NO_2_ affect fetal neurodevelopment might be different. These are proposed mechanisms through which NO_2_ may interfere with neuronal development.

Regarding ASD, our results on prenatal NO_2_ exposure are consistent with the associations reported in previous studies [43,56]. These previous studies reported that during all pregnancy periods, prenatal NO_2_ exposure was associated with the risk of ASD. On the other hand, Gong et al. reported that prenatal NO_2_ exposure is not associated with the risk of ASD [57,58]. However, these studies reported that the average prenatal NO_2_ level of exposure was around 14 μg/m^3^ to 20 μg/m^3^ or 5.4 μg/m^3^ to 12.7 μg/m^3^ in the observation period. These levels of NO_2_ were found to be lower than those of studies showing an association between NO_2_ and ASD (Wang et al., 24 μg/m^3^, Volk et al., 32.24 μg/m^3^) or this study (46.20 μg/m^3^).

Regarding epilepsy, there have been studies reporting that postnatal NO_2_ exposure is associated with the risk of epilepsy [59,60]. However, to the best of our knowledge, this study is the first to demonstrate that prenatal NO_2_ exposure is associated with the risk of epilepsy. It is known that prenatal NO_2_ exposure can induce oxidative stress [61,62] and systemic inflammation [63]. Oxidative stress can induce the placenta to secrete factors detrimental to neurons and expose fetal brains to oxidative stress, thus adversely affecting neuronal development. Inflammation can expose a fetus to maternal immune activation and pro-inflammatory cytokines, which can adversely affect neurodevelopment [64,65,66,67]. In animal studies, it has been found that prenatal NO_2_ exposure can adversely affect neonatal behavioral development [68]. Michikawa et al. [69] suggested that NO_2_ affects the placenta by inducing inflammation, and this may be related to inflammation of the endometrium. Other studies have shown that maternal exposure to NO_2_ is significantly associated with placental DNA methylation levels known to affect fetal development [37] and with DNA methylations that are associated with apoptosis-related genes in cord blood cells [70]. These are proposed mechanisms through which NO_2_ may interfere with neuronal development.

This study demonstrated that high concentrations of lead (Pb) exposure during the late pregnancy period was associated with the birth of a child with ASD and that exposure to Pb during the early pregnancy period was associated with the birth of child with epilepsy. High concentrations of Pb have been observed in hair and nail samples from children with ASD [71,72]. Skogheim et al. [73], using maternal blood samples, also suggested that prenatal Pb exposure is associated with the risk of ASD. Regarding epilepsy, Sasmaz et al. [74] reported that Pb concentrations were significantly higher in the hair of epilepsy patients than in the healthy group. Other studies have reported that Pb exposure during early development is associated with cognitive deficits, as well as behavioral abnormalities [75]. In a zebrafish study, prenatal exposure to water-soluble fractions of Pb could induce autism-like behavior in larvae [76]. Chen et al. [77] reported that prenatal Pb exposure can induce neurobehavioral anomalies in mice. Microglia are the most important innate immune cells in the brain. Pb has been shown to activate inflammasome proteins associated with microglial activation [78] and trigger microglial activation, releasing inflammatory cytokines and neural apoptosis [79]. Pb is a neurotoxicant that can suppress brain plasticity in a critical period of neurodevelopment [80]. Prenatal Pb exposure can cross the placenta and accumulate in fetal tissues, threating the developing brain and adversely affecting placenta functions [81]. Pb has been associated with altered DNA methylation patterns, with some affected genes being related to neurodevelopment or cognitive function [82,83]. Pb can impact the brain through DNA methylation mechanisms as well as interactions with calcium-ion-dependent processes and oxidative damage [84].

This study demonstrated that exposure to high concentrations of cadmium (Cd) during the prenatal late pregnancy period was associated with the birth of a child with epilepsy. Cd might be released from the mother and transferred to the fetus via the placenta. High concentrations of Cd have been found in the hair of infants with mothers occupationally exposed to Cd [85]. Prenatal Cd exposure is known to affect infant growth and organ development [86]. In animal studies, it has been found that Cd can affect neural development [87,88]. Some studies have revealed that exposure to Cd in early pregnancy is related to cognition or ASD and ADHD [73,89]. However, Forns et al. [90] reported that prenatal exposure to Cd is not related to cognition.

There are several limitations of this study. First, ASD is known to develop from complex interactions between genetic and environmental risk factors [91]. However, genetic factors were not considered in the present study. Second, ASD and epilepsy are influenced by postnatal exposure to air pollution. However, the findings were not adjusted for postnatal exposure. Since the subjects of this study were infants, their exposure after birth was unlikely to have had a significant effect. Third, co-exposure to toxic metals has a synergistic effect. However, there was no adjustment for this effect. For example, Pb and mercury have been found to have synergistic negative effects on childhood cognitive ability and development [92]. Gorini et al. [93] also discussed the impacts of single-heavy-metal exposure and co-exposure to multiple metals on the development of ASD.

The strength of this study was that it demonstrated the association of prenatal exposure to heavy metals with ASD and epilepsy using air pollution data. Previous studies have studied the risk of prenatal exposure using the hair or nail of the child and the blood or hair of the mother. To the best of our knowledge, this study was the first to measure the prenatal risk of each heavy metal as an air pollutant. The results demonstrate a more direct association between heavy metals in air pollution and the risk of prenatal exposure to ASD and epilepsy.

## 5. Conclusions

The findings of this study suggest that exposure to SO_2_, NO_2_, and Pb during pregnancy can affect the development of neurologic disorders according to the timing of exposure, indicating that such exposure is related to fetal development. The relationships of ASD and epilepsy with air pollution identified in this study need to be further clarified through more personalized assessments and further epidemiological studies. In addition, research on the mechanisms of toxic substances is needed. All these efforts will further clarify the causal relationship between air pollution and the incidence of ASD and epilepsy.

## Figures and Tables

**Table 1:** Demographic and prenatal characteristics of the study subjects.

	Total	With Autism Spectrum Disorder	With Epilepsy
Maternal age (years) *†			
total	843,134	5493	3190
<20	68 (0.0)	0 (0.0)	1 (0.0)
20–30	70,501 (8.4)	460 (8.4)	246 (7.7)
30–40	616,720 (73.1)	3740 (68.1)	2182 (68.4)
40<	155,845 (18.5)	1293 (23.5)	761 (23.9)
Occupational status *			
Worked outside home	323,250 (38.3)	1945 (35.4)	1187 (37.2)
Household income *			
Low (40%)	292,469 (34.7)	1819 (33.1)	1095 (34.3)
Middle (40–95%)	531,455 (63.0)	3526 (64.2)	2006 (62.9)
High (95% <)	19,210 (2.3)	148 (2.7)	89 (2.8)
Infant sex *†			
Male	428,873 (51.2)	3934 (71.6)	1765 (55.3)
Season *†			
Winter	224,344 (26.6)	1850 (33.7)	1170 (36.7)
Spring	218,963 (26.0)	1332 (24.2)	716 (22.4)
Summer	204,840 (24.3)	1196 (21.8)	679 (21.3)
Fall	194,987 (23.1)	1115 (20.3)	625 (19.6)
Premature (27–36 weeks) *†			
<36 weeks	44,060 (5.3)	829 (15.1)	430 (13.5)
Multiple birth *			
twin (Z38.3–Z38.5)	18,734 (2.2)	193 (3.5)	77 (2.4)

Data are presented as the mean ± standard deviation and number (%) of patients. * *p* < 0.05 compared between groups with and without autism spectrum disorder. † *p* < 0.05 compared between groups with and without epilepsy.

**Table 2:** Summary statistics of air pollutants and heavy metals by case.

Air Pollutants and Heavy Metals	Mean	Median
PM10 (μg/m^3^)	45.35 ± 11.07	45.07 (36.67–52.68)
PM2.5 (μg/m^3^)	25.83 ± 6.17	25.33 (21.36–29.71)
SO_2_ (ppm))	0.005 ± 0.001	0.005 (0.004–0.005)
NO_2_ (ppm)	0.023 ± 0.007	0.023 (0.018–0.028)
O_3_ (ppm)	0.028 ± 0.010	0.028 (0.020–0.035)
CO (ppm)	0.493 ± 0.110	0.468 (0.412–0.575)
Pb (μg/m^3^)	0.024 ± 0.012	0.022 (0.015–0.033)
Cd (μg/m^3^)	0.001 ± 0.001	0.001 (0.001–0.001)
Cr (μg/m^3^)	0.005 ± 0.004	0.004 (0.002–0.006)
Cu (μg/m^3^)	0.025 ± 0.018	0.021 (0.013–0.034)
Mn (μg/m^3^)	0.032 ± 0.017	0.029 (0.020–0.040)
Fe (μg/m^3^)	0.672 ± 0.345	0.650 (0.429–0.838)
Ni (μg/m^3^)	0.005 ± 0.003	0.004 (0.003–0.006)
As (μg/m^3^)	0.004 ± 0.003	0.003 (0.002–0.005)

Data are presented as the mean ± standard deviation and median (25–75%).

**Table 3:** Adjusted a odds ratio and 95% CI of autism spectrum disorder or epilepsy and exposure to air pollutants and heavy metals according to the stage of pregnancy in a single-pollutant model.

		With Autism Spectrum Disorder	With Epilepsy
		OR (95% CI)	OR (95% CI)
PM10	1st stage	0.992 (0.988–0.996)	0.992 (0.987–0.998)
	2nd stage	1.023 (1.019–1.027)	1.021 (1.016–1.027)
	3rd stage	1.022 (1.018–1.026)	1.020 (1.015–1.026)
	total	1.023 (1.017–1.029)	1.023 (1.015–1.030)
PM2.5	1st stage	0.995 (0.989–1.003)	0.996 (0.987–1.005)
	2nd stage	1.036 (1.029–1.043)	1.033 (1.024–1.043)
	3rd stage	1.039 (1.031–1.046)	1.033 (1.024–1.043)
	total	1.048 (1.037–1.059)	1.047 (1.033–1.061)
SO_2_	1st stage	2.843 (2.240–3.609)	2.900 (2.129–3.950)
	2nd stage	4.149 (3.183–5.408)	3.443 (2.434–4.871)
	3rd stage	5.060 (3.819–6.703)	5.670 (3.920–8.202)
	total	6.060 (4.449–8.256)	6.039 (4.033–9.041)
NO_2_	1st stage	1.281 (1.222–1.343)	1.485 (1.396–1.580)
	2nd stage	1.463 (1.396–1.533)	1.587 (1.492–1.688)
	3rd stage	1.394 (1.329–1.462)	1.577 (1.481–1.680)
	total	1.439 (1.367–1.515)	1.669 (1.559–1.786)
O_3_	1st stage	0.719 (0.679–0.761)	0.679 (0.629–0.732)
	2nd stage	0.764 (0.723–0.807)	0.716 (0.666–0.770)
	3rd stage	0.862 (0.813–0.914)	0.653 (0.603–0.707)
	total	0.572 (0.522–0.626)	0.404 (0.358–0.457)
CO	1st stage	1.008 (1.004–1.012)	1.014 (1.009–1.019)
	2nd stage	1.022 (1.018–1.026)	1.025 (1.020–1.030)
	3rd stage	1.021 (1.017–1.025)	1.032 (1.026–1.037)
	total	1.027 (1.022–1.033)	1.040 (1.033–1.047)
Pb	1st stage	1.097 (1.069–1.126)	1.147 (1.108–1.186)
	2nd stage	1.088 (1.060–1.117)	1.101 (1.063–1.139)
	3rd stage	1.139 (1.110–1.169)	1.164 (1.125–1.205)
	total	1.143 (1.109–1.178)	1.181 (1.136–1.228)
Cd	1st stage	2.038 (1.350–3.076)	3.045 (1.789–5.181)
	2nd stage	1.874 (1.316–2.667)	1.750 (1.101–2.782)
	3rd stage	2.458 (1.815–3.328)	3.288 (2.263–4.777)
	total	3.287 (2.113–5.113)	5.389 (3.061–9.487)
Cr	1st stage	1.075 (0.980–1.180)	1.059 (0.936–1.197)
	2nd stage	1.121 (1.020–1.233)	1.054 (0.929–1.195)
	3rd stage	1.102 (0.995–1.221)	1.122 (0.982–1.282)
	total	1.153 (1.026–1.297)	1.112 (0.954–1.297)
Cu	1st stage	1.028 (1.010–1.046)	1.071 (1.047–1.094)
	2nd stage	1.045 (1.026–1.064)	1.088 (1.063–1.114)
	3rd stage	1.059 (1.039–1.078)	1.103 (1.077–1.129)
	total	1.052 (1.031–1.073)	1.109 (1.080–1.137)
Mn	1st stage	1.016 (0.997–1.036)	1.003 (0.978–1.029)
	2nd stage	1.042 (1.023–1.061)	1.023 (0.999–1.048)
	3rd stage	1.031 (1.012–1.051)	1.031 (1.005–1.056)
	total	1.036 (1.014–1.058)	1.024 (0.996–1.054)
Fe	1st stage	1.001 (1.000–1.002)	1.003 (1.001–1.004)
	2nd stage	1.004 (1.003–1.005)	1.005 (1.004–1.007)
	3rd stage	1.004 (1.003–1.005)	1.004 (1.003–1.006)
	total	1.004 (1.003–1.005)	1.006 (1.004–1.007)
Ni	1st stage	1.054 (0.945–1.176)	1.020 (0.883–1.177)
	2nd stage	1.202 (1.078–1.340)	1.191 (1.032–1.374)
	3rd stage	1.394 (1.248–1.558)	1.296 (1.120–1.501)
	total	1.302 (1.144–1.481)	1.248 (1.054–1.479)
As	1st stage	1.565 (1.390–1.763)	1.311 (1.118–1.537)
	2nd stage	1.912 (1.700–2.152)	1.481 (1.260–1.741)
	3rd stage	1.660 (1.488–1.852)	1.687 (1.461–1.947)
	total	2.622 (2.222–3.095)	2.039 (1.642–2.532)

Logistic regression model adjusted for maternal age, education, infant sex, season of conception, and household income.

**Table 4:** Adjusted odds ratios and 95% CIs for autism spectrum disorder and exposure to air pollutants and heavy metals according to the stage of pregnancy in a four-pollutant model.

		With Autism Spectrum Disorder
SO_2_ + NO_2_ + Pb + Cd		OR (95%CI)
SO_2_	Total	3.288 (2.306–4.687)
	1st stage	1.770 (1.338–2.342)
	2nd stage	2.128 (1.557–2.909)
	3rd stage	2.723 (1.971–3.761)
NO_2_	Total	1.322 (1.244–1.403)
	1st stage	1.233 (1.165–1.304)
	2nd stage	1.421 (1.346–1.501)
	3rd stage	1.267 (1.197–1.341)
Pb	Total	1.079 (1.017–1.145)
	1st stage	1.041 (0.993–1.092)
	2nd stage	0.980 (0.936–1.026)
	3rd stage	1.063 (1.019–1.110)
Cd	Total	0.441 (0.184–1.057)
	1st stage	1.233 (1.165–1.304)
	2nd stage	1.421 (1.346–1.501)
	3rd stage	1.267 (1.197–1.341)

Logistic regression model was adjusted for maternal age, education, infant sex, season of conception, and household income.

**Table 5:** Adjusted odds ratio and 95% CIs for epilepsy and exposure to air pollutants and heavy metals according to the stage of pregnancy in a five-pollutant model.

		With Epilepsy
SO_2_ + NO_2_ + Pb + Cd + As		OR (95%CI)
SO_2_	Total	3.702 (2.25–6.089)
	1st stage	2.106 (1.403–3.163)
	2nd stage	1.648 (1.061–2.560)
	3rd stage	2.897 (1.855–4.522)
NO_2_	Total	1.869 (1.696–2.059)
	1st stage	1.542 (1.422–1.672)
	2nd stage	1.680 (1.553–1.817)
	3rd stage	1.548 (1.427–1.681)
Pb	Total	1.064 (0.983–1.152)
	1st stage	1.109 (1.043–1.179)
	2nd stage	1.039 (0.976–1.107)
	3rd stage	1.031 (0.974–1.092)
Cd	Total	2.591 (0.738–9.102)
	1st stage	0.857 (0.328–2.243)
	2nd stage	0.757 (0.308–1.859)
	3rd stage	2.193 (1.074–4.477)
As	Total	0.273 (0.187–0.399)
	1st stage	0.461 (0.361–0.589)
	2nd stage	0.568 (0.436–0.741)
	3rd stage	0.613 (0.480–0.783)

Logistic regression model was adjusted for maternal age, education, infant sex, season of conception, and household income.

## Data Availability

Not applicable.

## References

[1] Cohen A.J., Brauer M., Burnett R., Anderson H.R., Frostad J., Estep K., Balakrishnan K., Brunekreef B., Dandona L., Dandona R. (2017). Estimates and 25-year trends of the global burden of disease attributable to ambient air pollution: An analysis of data from the Global Burden of Diseases Study 2015. Lancet.

[2] Wang R., Liu J., Qin Y., Chen Z., Li J., Guo P., Shan L., Li Y., Hao Y., Jiao M. (2023). Global attributed burden of death for air pollution: Demographic decomposition and birth cohort effect. Sci. Total Environ..

[3] McDuffie E., Martin R., Yin H., Brauer M. (2021). Global Burden of Disease from Major Air Pollution Sources (GBD MAPS): A Global Approach. Res. Rep. Health Eff. Inst..

[4] Ju M.J., Oh J., Choi Y.H. (2021). Changes in air pollution levels after COVID-19 outbreak in Korea. Sci. Total Environ..

[5] Yang N., Zhang Z., Xue B., Ma J., Chen X., Lu C. (2018). Economic growth and pollution emission in China: Structural path analysis. Sustainability.

[6] Ngarambe J., Joen S.J., Han C.H., Yun G.Y. (2021). Exploring the relationship between particulate matter, CO, SO(2), NO(2), O(3) and urban heat island in Seoul, Korea. J. Hazard. Mater..

[7] Guaman M., Roberts-Semple D., Aime C., Shin J., Akinremi A. (2022). Traffic Density and Air Pollution: Spatial and Seasonal Variations of Nitrogen Dioxide and Ozone in Jamaica, New York. Atmosphere.

[8] Choi E., Yi S.M., Lee Y.S., Jo H., Baek S.O., Heo J.B. (2022). Sources of airborne particulate matter-bound metals and spatial-seasonal variability of health risk potentials in four large cities, South Korea. Environ. Sci. Pollut. Res. Int..

[9] Valavanidis A., Fiotakis K., Vlahogianni T., Bakeas E.B., Triantafillaki S., Paraskevopoulou V., Dassenakis M. (2006). Characterization of atmospheric particulates, particle-bound transition metals and polycyclic aromatic hydrocarbons of urban air in the centre of Athens (Greece). Chemosphere.

[10] Querol X., Viana M., Alastuey A., Amato F., Moreno T., Castillo S., Pey J., Rosa J.d.l., Campa A.S.d.l., Artíñano B. (2007). Source origin of trace elements in PM from regional background, urban and industrial sites of Spain. Atmos. Environ..

[11] Meng X., Ma Y., Chen R., Zhou Z., Chen B., Kan H. (2013). Size-fractionated particle number concentrations and daily mortality in a Chinese city. Environ. Health Perspect..

[12] Samoli E., Atkinson R.W., Analitis A., Fuller G.W., Beddows D., Green D.C., Mudway I.S., Harrison R.M., Anderson H.R., Kelly F.J. (2016). Differential health effects of short-term exposure to source-specific particles in London, U.K. Environ. Int..

[13] Alimohammadi H., Fakhri S., Derakhshanfar H., Hosseini-Zijoud S.M., Safari S., Hatamabadi H.R. (2016). The Effects of Air Pollution on Ischemic Stroke Admission Rate. Chonnam Med. J..

[14] Simoncic V., Enaux C., Deguen S., Kihal-Talantikite W. (2020). Adverse Birth Outcomes Related to NO(2) and PM Exposure: European Systematic Review and Meta-Analysis. Int. J. Environ. Res. Public Health.

[15] Lertxundi A., Andiarena A., Martinez M.D., Ayerdi M., Murcia M., Estarlich M., Guxens M., Sunyer J., Julvez J., Ibarluzea J. (2019). Prenatal exposure to PM(2.5) and NO(2) and sex-dependent infant cognitive and motor development. Environ. Res..

[16] Nemmar A., Hoylaerts M.F., Hoet P.H., Nemery B. (2004). Possible mechanisms of the cardiovascular effects of inhaled particles: Systemic translocation and prothrombotic effects. Toxicol. Lett..

[17] Liu Z., Wang W., Cao F., Liu S., Zou X., Li G., Yang H., Jiao Y. (2018). Number 2 Feibi Recipe Reduces PM2.5-Induced Lung Injury in Rats. Evid. Based Complement Alternat. Med..

[18] Piao C.H., Fan Y., Nguyen T.V., Shin H.S., Kim H.T., Song C.H., Chai O.H. (2021). PM(2.5) Exacerbates Oxidative Stress and Inflammatory Res.ponse through the Nrf2/NF-kappaB Signaling Pathway in OVA-Induced Allergic Rhinitis Mouse Model. Int. J. Mol. Sci..

[19] Thornburg K.L., Kolahi K., Pierce M., Valent A., Drake R., Louey S. (2016). Biological features of placental programming. Placenta.

[20] Siddika N., Balogun H.A., Amegah A.K., Jaakkola J.J. (2016). Prenatal ambient air pollution exposure and the risk of stillbirth: Systematic review and meta-analysis of the empirical evidence. Occup. Environ. Med..

[21] Enkhmaa D., Warburton N., Javzandulam B., Uyanga J., Khishigsuren Y., Lodoysamba S., Enkhtur S., Warburton D. (2014). Seasonal ambient air pollution correlates strongly with spontaneous abortion in Mongolia. BMC Pregnancy Childbirth.

[22] Wu A., Ying Z., Gomez-Pinilla F. (2004). The interplay between oxidative stress and brain-derived neurotrophic factor modulates the outcome of a saturated fat diet on synaptic plasticity and cognition. Eur. J. Neurosci..

[23] Eyre H., Baune B.T. (2012). Neuroplastic changes in depression: A role for the immune system. Psychoneuroendocrinology.

[24] Goel R., Bhat S.A., Hanif K., Nath C., Shukla R. (2018). Angiotensin II Receptor Blockers Attenuate Lipopolysaccharide-Induced Memory Impairment by Modulation of NF-kappaB-Mediated BDNF/CREB Expression and Apoptosis in Spontaneously Hypertensive Rats. Mol. Neurobiol..

[25] Saenen N.D., Plusquin M., Bijnens E., Janssen B.G., Gyselaers W., Cox B., Fierens F., Molenberghs G., Penders J., Vrijens K. (2015). In Utero Fine Particle Air Pollution and Placental Expression of Genes in the Brain-Derived Neurotrophic Factor Signaling Pathway: An ENVIRONAGE Birth Cohort Study. Environ. Health Perspect..

[26] Wei H., Liang F., Meng G., Nie Z., Zhou R., Cheng W., Wu X., Feng Y., Wang Y. (2016). Redox/methylation mediated abnormal DNA methylation as regulators of ambient fine particulate matter-induced neurodevelopment related impairment in human neuronal cells. Sci. Rep..

[27] Chen M., Li B., Sang N. (2017). Particulate matter (PM(2.5)) exposure season-dependently induces neuronal apoptosis and synaptic injuries. J. Environ. Sci. (China).

[28] Folstein S., Rutter M. (1977). Infantile autism: A genetic study of 21 twin pairs. J. Child Psychol. Psychiatry.

[29] Steffenburg S., Gillberg C., Hellgren L., Andersson L., Gillberg I.C., Jakobsson G., Bohman M. (1989). A twin study of autism in Denmark, Finland, Iceland, Norway and Sweden. J. Child Psychol. Psychiatry.

[30] Jeste S.S., Geschwind D.H. (2014). Disentangling the heterogeneity of autism spectrum disorder through genetic findings. Nat. Rev. Neurol..

[31] Hallmayer J., Cleveland S., Torres A., Phillips J., Cohen B., Torigoe T., Miller J., Fedele A., Collins J., Smith K. (2011). Genetic heritability and shared environmental factors among twin pairs with autism. Arch. Gen. Psychiatry.

[32] Sandin S., Lichtenstein P., Kuja-Halkola R., Larsson H., Hultman C.M., Reichenberg A. (2014). The familial risk of autism. JAMA.

[33] Sarkisova K., van Luijtelaar G. (2022). The impact of early-life environment on absence epilepsy and neuropsychiatric comorbidities. IBRO Neurosci. Rep..

[34] Kannan S., Misra D.P., Dvonch J.T., Krishnakumar A. (2006). Exposures to airborne particulate matter and adverse perinatal outcomes: A biologically plausible mechanistic framework for exploring potential effect modification by nutrition. Environ. Health Perspect..

[35] Seltenrich N. (2016). PM2.5 Exposure and Intrauterine Inflammation: A Possible Mechanism for Preterm and Underweight Birth. Environ. Health Perspect..

[36] van den Hooven E.H., Pierik F.H., de Kluizenaar Y., Hofman A., van Ratingen S.W., Zandveld P.Y., Russcher H., Lindemans J., Miedema H.M., Steegers E.A. (2012). Air pollution exposure and markers of placental growth and function: The generation R study. Environ. Health Perspect..

[37] Abraham E., Rousseaux S., Agier L., Giorgis-Allemand L., Tost J., Galineau J., Hulin A., Siroux V., Vaiman D., Charles M.A. (2018). Pregnancy exposure to atmospheric pollution and meteorological conditions and placental DNA methylation. Environ. Int..

[38] Hung T.H., Hsu T.Y., Tung T.H., Tsai C.C., Ou C.Y., Chung F.F., Wan G.H. (2021). The association between maternal exposure to outdoor air pollutants, inflammatory response, and birth weight in healthy women. Environ. Res..

[39] Cao Z., Meng L., Zhao Y., Liu C., Yang Y., Su X., Fu Q., Wang D., Hua J. (2019). Maternal exposure to ambient fine particulate matter and fetal growth in Shanghai, China. Environ. Health.

[40] Rich D.Q., Liu K., Zhang J., Thurston S.W., Stevens T.P., Pan Y., Kane C., Weinberger B., Ohman-Strickland P., Woodruff T.J. (2015). Differences in Birth Weight Associated with the 2008 Beijing Olympics Air Pollution Reduction: Res.ults from a Natural Experiment. Environ. Health Perspect..

[41] Zhao Y., Wang P., Zhou Y., Xia B., Zhu Q., Ge W., Li J., Shi H., Xiao X., Zhang Y. (2021). Prenatal fine particulate matter exposure, placental DNA methylation changes, and fetal growth. Environ. Int..

[42] Gluckman P.D., Hanson M.A., Cooper C., Thornburg K.L. (2008). Effect of in utero and early-life conditions on adult health and disease. N. Engl. J. Med..

[43] Wang S.Y., Cheng Y.Y., Guo H.R., Tseng Y.C. (2021). Air Pollution during Pregnancy and Childhood Autism Spectrum Disorder in Taiwan. Int. J. Environ. Res. Public Health.

[44] Lin C.C., Yang S.K., Lin K.C., Ho W.C., Hsieh W.S., Shu B.C., Chen P.C. (2014). Multilevel analysis of air pollution and early childhood neurobehavioral development. Int. J. Environ. Res. Public Health.

[45] Yu T., Zhou L., Xu J., Kan H., Chen R., Chen S., Hua H., Liu Z., Yan C. (2021). Effects of prenatal exposures to air sulfur dioxide/nitrogen dioxide on toddler neurodevelopment and effect modification by ambient temperature. Ecotoxicol. Environ. Saf..

[46] Yargicoglu P., Agar A., Gumuslu S., Bilmen S., Oguz Y. (1999). Age-related alterations in antioxidant enzymes, lipid peroxide levels, and somatosensory-evoked potentials: Effect of sulfur dioxide. Arch. Environ. Contam. Toxicol..

[47] Kucukatay V., Agar A., Yargicoglu P., Gumuslu S., Aktekin B. (2003). Changes in somatosensory evoked potentials, lipid peroxidation, and antioxidant enzymes in experimental diabetes: Effect of sulfur dioxide. Arch. Environ. Health.

[48] Meng Z., Zhang B. (2003). Oxidative damage of sulfur dioxide inhalation on brains and livers of mice. Environ. Toxicol. Pharmacol..

[49] Yun Y., Li H., Li G., Sang N. (2010). SO2 inhalation modulates the expression of apoptosis-related genes in rat hippocampus via its derivatives in vivo. Inhal. Toxicol..

[50] Choi Y.J., Cho J., Hong Y.C., Lee D.W., Moon S., Park S.J., Lee K.S., Shin C.H., Lee Y.A., Kim B.N. (2023). DNA methylation is associated with prenatal exposure to sulfur dioxide and childhood attention-deficit hyperactivity disorder symptoms. Sci. Rep..

[51] Gunnison A.F., Benton A.W. (1971). Sulfur dioxide: Sulfite. Interaction with mammalian serum and plasma. Arch. Environ. Health.

[52] Gunnison A.F. (1981). Sulphite toxicity: A critical review of in vitro and in vivo data. Food Cosmet. Toxicol..

[53] Muller F., Massey V. (1969). Flavin-sulfite complexes and their structures. J. Biol. Chem..

[54] Kamogawa A., Fukui T. (1973). Inhibition of -glucan phosphorylase by bisulfite competition at the phosphate binding site. Biochim. Biophys. Acta.

[55] Liu Y., Li L., Xie J., Jiao X., Hu H., Zhang Y., Tao R., Tao F., Zhu P. (2021). Foetal 25-hydroxyvitamin D moderates the association of prenatal air pollution exposure with foetal glucolipid metabolism disorder and systemic inflammatory responses. Environ. Int..

[56] Volk H.E., Lurmann F., Penfold B., Hertz-Picciotto I., McConnell R. (2013). Traffic-related air pollution, particulate matter, and autism. JAMA Psychiatry.

[57] Gong T., Dalman C., Wicks S., Dal H., Magnusson C., Lundholm C., Almqvist C., Pershagen G. (2017). Perinatal Exposure to Traffic-Related Air Pollution and Autism Spectrum Disorders. Environ. Health Perspect..

[58] Gong T., Almqvist C., Bolte S., Lichtenstein P., Anckarsater H., Lind T., Lundholm C., Pershagen G. (2014). Exposure to air pollution from traffic and neurodevelopmental disorders in Swedish twins. Twin Res. Hum. Genet..

[59] Cheng J., Su H., Song J., Wang X. (2022). Short-term effect of air pollution on childhood epilepsy in eastern China: A space-time-stratified case-crossover and pooled analysis. Environ. Int..

[60] Cakmak S., Dales R.E., Vidal C.B. (2010). Air pollution and hospitalization for epilepsy in Chile. Environ. Int..

[61] Clemente D.B.P., Casas M., Janssen B.G., Lertxundi A., Santa-Marina L., Iniguez C., Llop S., Sunyer J., Guxens M., Nawrot T.S. (2017). Prenatal ambient air pollution exposure, infant growth and placental mitochondrial DNA content in the INMA birth cohort. Environ. Res..

[62] Decrue F., Gorlanova O., Salem Y., Vienneau D., de Hoogh K., Gisler A., Usemann J., Korten I., Nahum U., Sinues P. (2022). Increased Impact of Air Pollution on Lung Function in Preterm versus Term Infants: The BILD Study. Am. J. Respir. Crit. Care Med..

[63] Zhang B., Gong X., Han B., Chu M., Gong C., Yang J., Chen L., Wang J., Bai Z., Zhang Y. (2022). Ambient PM(2.5) exposures and systemic inflammation in women with early pregnancy. Sci. Total Environ..

[64] Gyllenhammer L.E., Rasmussen J.M., Bertele N., Halbing A., Entringer S., Wadhwa P.D., Buss C. (2022). Maternal Inflammation During Pregnancy and Offspring Brain Development: The Role of Mitochondria. Biol. Psychiatry Cogn. Neurosci. Neuroimaging.

[65] Barron A., McCarthy C.M., O’Keeffe G.W. (2021). Preeclampsia and Neurodevelopmental Outcomes: Potential Pathogenic Roles for Inflammation and Oxidative Stress?. Mol. Neurobiol..

[66] Valencia A., Moran J. (2001). Role of oxidative stress in the apoptotic cell death of cultured cerebellar granule neurons. J. Neurosci. Res..

[67] Xu W., Chi L., Row B.W., Xu R., Ke Y., Xu B., Luo C., Kheirandish L., Gozal D., Liu R. (2004). Increased oxidative stress is associated with chronic intermittent hypoxia-mediated brain cortical neuronal cell apoptosis in a mouse model of sleep apnea. Neuroscience.

[68] Singh J. (1988). Nitrogen dioxide exposure alters neonatal development. Neurotoxicology.

[69] Michikawa T., Morokuma S., Yamazaki S., Fukushima K., Kato K., Nitta H. (2016). Exposure to air pollutants during the early weeks of pregnancy, and placenta praevia and placenta accreta in the western part of Japan. Environ. Int..

[70] Park J., Kim W.J., Kim J., Jeong C.Y., Park H., Hong Y.C., Ha M., Kim Y., Won S., Ha E. (2022). Prenatal Exposure to Traffic-Related Air Pollution and the DNA Methylation in Cord Blood Cells: MOCEH Study. Int. J. Environ. Res. Public Health.

[71] Zhai Q., Cen S., Jiang J., Zhao J., Zhang H., Chen W. (2019). Disturbance of trace element and gut microbiota profiles as indicators of autism spectrum disorder: A pilot study of Chinese children. Environ. Res..

[72] Lakshmi Priya M.D., Geetha A. (2011). Level of trace elements (copper, zinc, magnesium and selenium) and toxic elements (lead and mercury) in the hair and nail of children with autism. Biol. Trace Elem. Res..

[73] Skogheim T.S., Weyde K.V.F., Engel S.M., Aase H., Suren P., Oie M.G., Biele G., Reichborn-Kjennerud T., Caspersen I.H., Hornig M. (2021). Metal and essential element concentrations during pregnancy and associations with autism spectrum disorder and attention-deficit/hyperactivity disorder in children. Environ. Int..

[74] Sasmaz S., Uz E., Pinar T., Vural H.s., Eiri M.c., Ilihan A., Akyol m. (2003). Hair lead and cadmium concentrations in patients with epilepsy and migraine. Neurosci. Res. Commun..

[75] Eubig P.A., Aguiar A., Schantz S.L. (2010). Lead and PCBs as risk factors for attention deficit/hyperactivity disorder. Environ. Health Perspect..

[76] Wang Y., Zhong H., Wang C., Gao D., Zhou Y., Zuo Z. (2016). Maternal exposure to the water soluble fraction of crude oil, lead and their mixture induces autism-like behavioral deficits in zebrafish (Danio rerio) larvae. Ecotoxicol. Environ. Saf..

[77] Chen Y., Liu S., Xu H., Zheng H., Bai C., Pan W., Zhou H., Liao M., Huang C., Dong Q. (2019). Maternal exposure to low dose BDE209 and Pb mixture induced neurobehavioral anomalies in C57BL/6 male offspring. Toxicology.

[78] Su P., Wang D., Cao Z., Chen J., Zhang J. (2021). The role of NLRP3 in lead-induced neuroinflammation and possible underlying mechanism. Environ. Pollut..

[79] Liu J.T., Chen B.Y., Zhang J.Q., Kuang F., Chen L.W. (2015). Lead exposure induced microgliosis and astrogliosis in hippocampus of young mice potentially by triggering TLR4-MyD88-NFkappaB signaling cascades. Toxicol. Lett..

[80] Smith M.R., Yevoo P., Sadahiro M., Austin C., Amarasiriwardena C., Awawda M., Arora M., Dudley J.T., Morishita H. (2018). Integrative bioinformatics identifies postnatal lead (Pb) exposure disrupts developmental cortical plasticity. Sci. Rep..

[81] Gundacker C., Hengstschlager M. (2012). The role of the placenta in fetal exposure to heavy metals. Wien. Med. Wochenschr..

[82] Dou J.F., Farooqui Z., Faulk C.D., Barks A.K., Jones T., Dolinoy D.C., Bakulski K.M. (2019). Perinatal Lead (Pb) Exposure and Cortical Neuron-Specific DNA Methylation in Male Mice. Genes.

[83] Montrose L., Faulk C., Francis J., Dolinoy D.C. (2017). Perinatal lead (Pb) exposure results in sex and tissue-dependent adult DNA methylation alterations in murine IAP transposons. Environ. Mol. Mutagen.

[84] Sanders T., Liu Y., Buchner V., Tchounwou P.B. (2009). Neurotoxic effects and biomarkers of lead exposure: A review. Rev. Environ. Health.

[85] Huel G., Everson R.B., Menger I. (1984). Increased hair cadmium in newborns of women occupationally exposed to heavy metals. Environ. Res..

[86] Lin C.M., Doyle P., Wang D., Hwang Y.H., Chen P.C. (2011). Does prenatal cadmium exposure affect fetal and child growth?. Occup. Environ. Med..

[87] Grover C.A., Nation J.R., Reynolds K.M., Benzick A.E., Bratton G.R., Rowe L.D. (1991). The effects of cadmium on ethanol self-administration using a sucrose-fading procedure. Neurotoxicology.

[88] Pelletier M.R., Satinder K.P. (1991). Low-level cadmium exposure increases one-way avoidance in juvenile rats. Neurotoxicol. Teratol..

[89] Kim Y., Ha E.H., Park H., Ha M., Kim Y., Hong Y.C., Kim E.J., Kim B.N. (2013). Prenatal lead and cadmium co-exposure and infant neurodevelopment at 6 months of age: The Mothers and Children’s Environ.mental Health (MOCEH) study. Neurotoxicology.

[90] Forns J., Fort M., Casas M., Caceres A., Guxens M., Gascon M., Garcia-Esteban R., Julvez J., Grimalt J.O., Sunyer J. (2014). Exposure to metals during pregnancy and neuropsychological development at the age of 4 years. Neurotoxicology.

[91] Lai M.C., Lombardo M.V., Baron-Cohen S. (2014). Autism. Lancet.

[92] Chisolm J.J. (1974). The susceptibility of the fetus and child to chemical pollutants. Heavy metal exposures: Toxicity from metal-metal interactions, and behavioral effects. Pediatrics.

[93] Gorini F., Muratori F., Morales M.A. (2014). The Role of Heavy Metal Pollution in Neurobehavioral Disorders: A Focus on Autism. Rev. J. Autism Dev. Disord..

